# Tunable Microwave Dielectric Properties of Ca_0.6_La_0.8/3_TiO_3_ and Ca_0.8_Sm_0.4/3_TiO_3_-Modified (Mg_0.6_Zn_0.4_)_0.95_Ni_0.05_TiO_3_ Ceramics with a Near-Zero Temperature Coefficient

**DOI:** 10.3390/molecules26164715

**Published:** 2021-08-04

**Authors:** Chung-Long Pan, Chun-Hsu Shen, Shih-Hung Lin, Qi-Zi Lin

**Affiliations:** 1 Department of Electrical Engineering, I-Shou University, No. 1, Sec. 1, Syuecheng Rd., Dashu District, Kaohsiung City 84001, Taiwan; ptl@isu.edu.tw; 2 Department of Electronic Engineering, National Yunlin University of Science and Technology, Section 3, 123 University Road, Douliou, Yunlin 64002, Taiwan; jameschs@yuntech.edu.tw (C.-H.S.); M10913071@yuntech.edu.tw (Q.-Z.L.)

**Keywords:** temperature coefficient, dielectric properties, wireless communication, near-zero temperature coefficient

## Abstract

The microstructures and microwave dielectric properties of (Mg_0.6_Zn_0.4_)_0.95_Ni_0.05_TiO_3_ with Ca_0.6_La_0.8/3_TiO_3_ and Ca_0.8_Sm_0.4/3_TiO_3_ additions prepared by the solid-state method has been investigated. The crystallization and microstructures of these two mixed dielectrics were checked by XRD, EDX, BEI, and SEM to demonstrate two phase systems. Furthermore, the tunable dielectric properties can be achieved by adjusting the amounts of Ca_0.6_La_0.8/3_TiO_3_ and Ca_0.8_Sm_0.4/3_TiO_3_ additions, respectively. After optimization of processed parameters, a new dielectric material system 0.88(Mg_0.6_Zn_0.4_)_0.95_Ni_0.05_TiO_3_-0.12Ca_0.6_La_0.8/3_TiO_3_ possesses a permittivity (ε_r_) of 24.7, a Q*f* value of 106,000 (GHz), and a τ*_f_* value of 3.8 (ppm/°C), with sintering temperature at 1225 °C for 4 h. This dielectric system with a near-zero temperature coefficient and appropriate microwave properties revealed a high potential for high-quality substrates adopted in wireless communication devices.

## 1. Introduction

With the ever-growing requirements of wireless communication devices and systems, there is a rapidly evolving lack of high-performance microwave circuits, receivers, transceivers, etc., to address the numerous 5G wireless communications technologies. Therefore, the utilization of dielectric ceramics with high permittivity (ε_r_) and low dielectric loss has attracted more and more attention. In industrial applications, dielectric materials require the consideration of three parameters: an applicable relative permittivity (ε_r_), a high-quality factor (Q*f*), and a near-zero temperature coefficient of resonance frequency (τ*_f_*) [1,2,3,4,5,6,7]. Dielectric materials satisfied with these conditions demonstrated a reduction in component size and dielectric loss; conversely, the component characteristics are not affected by external temperature changes [8,9,10].

The MgTiO_3_-based ceramics were documented as an ilmenite-type structure and showed an excellent dielectric performance in high-frequency applications [11]. To upgrade the dielectric performances of MgTiO_3_-based ceramics, some studies focus on substituting Mg with M^2+^ (M^2+^ = Co, Ni, and Zn) and the (Mg_0.95_M^2+^_0.05_)TiO_3_ ceramics preserve the ilmenite-type structure [11,12]. Shen et al. [13] first reported Mg_0.95_Ni_0.05_TiO_3_ with a Q*f* of 192,000 (GHz), ε_r_ ~17.35, and τ*_f_* of –47 (ppm/°C) for the samples sintered at 1350 °C and 4 h. The main disadvantage of (Mg_0.95_M^2+^_0.05_)TiO_3_ ceramics is their high negative τ*_f_* and, hence, difficulty to be practically utilized in microwave applications. Therefore, some researchers improved the microwave dielectric properties of (Mg_0.95_M^2+^_0.05_)TiO_3_ by mixing τ*_f_* compensator [14]. With an appropriate stoichiometric of τ*_f_* compensator additions, the mixture demonstrated near-zero τ*_f_* with an appropriate Q*f* value and permittivity. For example, it was found that the composition of 0.95MgTiO_3_-0.05CaTiO_3_ ceramics has a zero τ*_f_*. Furthermore, Ca_0.6_La_0.8/3_TiO_3_ and Ca_0.8_Sm_0.4/3_TiO_3_ were added in Mg_0.95_Ni_0.05_TiO_3_ to obtain near-zero τ*_f_* mixtures for practical applications in microwave components [15,16]. In addition, with the further substitution of Mg^2+^ (0.72 Å) by Zn^2+^(0.82 Å), the (Mg_0.6_Zn_0.4_)_0.95_Ni_0.05_TiO_3_ ceramics were also synthesized by a traditional solid-state method that had been reported to possess Q*f* ~ 165,000 (GHz), ε_r_ of 19.3, and τ*_f_* of −65.4 (ppm/°C) under sintering at 1200 °C/4 h by Lin et al. [17]. However, to our best knowledge, the microwave dielectric properties of (Mg_0.6_Zn_0.4_)_0.95_Ni_0.05_TiO_3_ with any τ*_f_* compensator to adjust τ*_f_* approaching zero have not been studied. The thermal budget of (Mg_0.6_Zn_0.4_)_0.95_Ni_0.05_TiO_3_ (1200 °C * 4 h) showed an obvious reduction compared to Mg_0.95_Ni_0.05_TiO_3_ (1350 °C * 4 h). Therefore, the study of low thermal budget (Mg_0.6_Zn_0.4_)_0.95_Ni_0.05_TiO_3_-based ceramics with near-zero temperature coefficient and satisfied microwave dielectric properties via τ*_f_* compensators additions is crucial for industrial applications.

In this work, two state-of-the-art τ*_f_* compensators, Ca_0.6_La_0.8/3_TiO_3_ (ε_r_ ~ 117.4, Q*f* ~ 13,375 GHz, and τ*_f_* ~ 217.2 ppm/°C) and Ca_0.8_Sm_0.4/3_TiO_3_ (ε_r_ ~ 120, Q*f* ~ 13,800 GHz, and τ*_f_* ~ 400 ppm/°C), were chosen to mix with (Mg_0.6_Zn_0.4_)_0.95_Ni_0.05_TiO_3_ to characterize their dielectric properties, respectively. The mixtures of x(Mg_0.6_Zn_0.4_)_0.95_Ni_0.05_TiO_3_-(1 − x) Ca_0.6_La_0.8/3_TiO_3_/Ca_0.8_Sm_0.4/3_TiO_3_, which clarify the enhancement of its temperature coefficient characteristics for the achievement of a near-zero τ*_f_* point. Densification, X-ray diffraction patterns, and microstructures were employed to analyze the physical properties of mixtures. The correlation between physical properties and microwave properties was investigated in detail and depth. Furthermore, the comparisons of thermal budget and microwave dielectric properties between (Mg_0.6_Zn_0.4_)_0.95_Ni_0.05_TiO_3_ (1200 °C * 4 h) and Mg_0.95_Ni_0.05_TiO_3_ (1350 °C * 4 h) with τ*_f_* compensators were presented.

## 2. Results and Discussion

### 2.1. Physical Investigation

**Figure 1:**
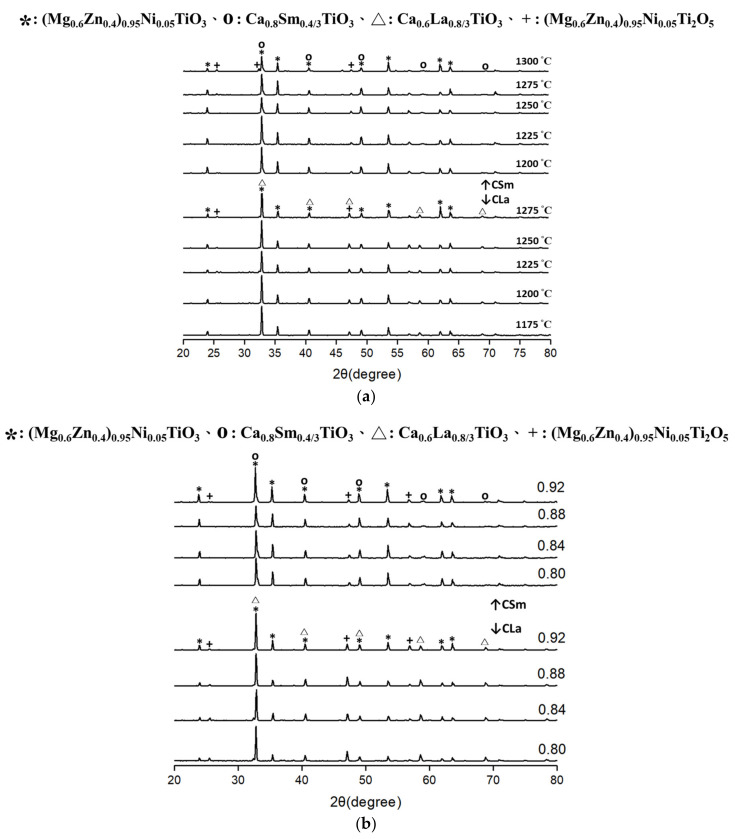
X-ray diffraction illustrations of (**a**) 0.88(Mg_0.6_Zn_0.4_)_0.95_Ni_0__.__05_TiO_3_-0.12 Ca_0__.6_La_0__.8__/3_TiO_3_/Ca_0__.8_Sm_0__.4__/3_TiO_3_ ceramics sintered at various temperatures for 4 h, (**b**) x(Mg_0.6_Zn_0.4_)_0.95_Ni_0__.__05_TiO_3_-(1 − x)Ca_0__.6_La_0__.8__/3_TiO_3_ sintered at 1225 °C for 4 h /Ca_0__.__8_Sm_0__.__4__/__3_TiO_3_ sintered at 1250 °C for 4 h with various x values.

The XRD analysis for x(Mg_0.6_Zn_0.4_)_0.95_Ni_0.__05_TiO_3_-(1–x)Ca_0.6_La_0.8/3_TiO_3_/Ca_0.8_Sm_0.4/3_TiO_3_ (hereafter referred to as xMZNT-(1–x)CLa/CSm) with x = 0.88 sintered at 1175 °C–1300 °C for 4 h and sintered at 1225 °C (Ca_0.6_La_0.8/3_TiO_3_)/1250 °C (Ca_0.8_Sm_0.4/3_TiO_3_) for 4 h with various x values, are illustrated in Figure 1a,b, respectively. The X-ray patterns indicated the presence of (Mg_0.6_Zn_0.4_)_0.95_Ni_0.05_TiO_3_ signals as the primary crystalline phase with a less minor phase of Ca_0.6_La_0.8/3_TiO_3_ (ICDD-PDF #22-0153) or Ca_0.8_Sm_0__.4/3_TiO_3_(ICDD-PDF #78-1371) [18,19], and the second phase of (Mg_0.6_Zn_0.4_)_0.95_Ni_0.05_Ti_2_O_5_ (which can be referred to as MgTi_2_O_5_). It was reported that the crystal structures of (Mg_0.6_Zn_0.4_)_0.95_Ni_0.05_TiO_3_ are hexagonal, and those of Ca_0.6_La_0.8/3_TiO_3_ and Ca_0.8_Sm_0.4/3_TiO_3_ are cubic. (Mg_0.6_Zn_0.4_)_0.95_Ni_0.05_Ti_2_O_5_ with the orthorhombic crystal structure (ICDD-PDF #00009–0016), usually formed as an intermediate phase, was identified and difficult to remove from the MgTiO_3_-based sample composed by the traditional mixed oxide route [20,21,22]. The composition of the second phase (Mg_0.6_Zn_0.4_)_0.95_Ni_0.05_Ti_2_O_5,_ which might diminish the *Qf* values of the specimen [22], has primarily resulted from the loss of ignition (LOI) of the raw powder MgO. The following reaction (Equation (1)) may explain this phenomenon:

2(Mg_0.6_Zn_0.4_)_0.95_Ni_0.05_TiO_3_ → (Mg_0.6_Zn_0.4_)_0.95_Ni_0.05_Ti_2_O_5_ + (Mg_0.6_Zn_0.4_)_0.95_Ni_0.05_O (1)

X-ray diffraction results of the xMZNT-(1 − x)CLa/CSm systems demonstrated no significant change with varying sintering temperature and x value.

**Figure 2:**
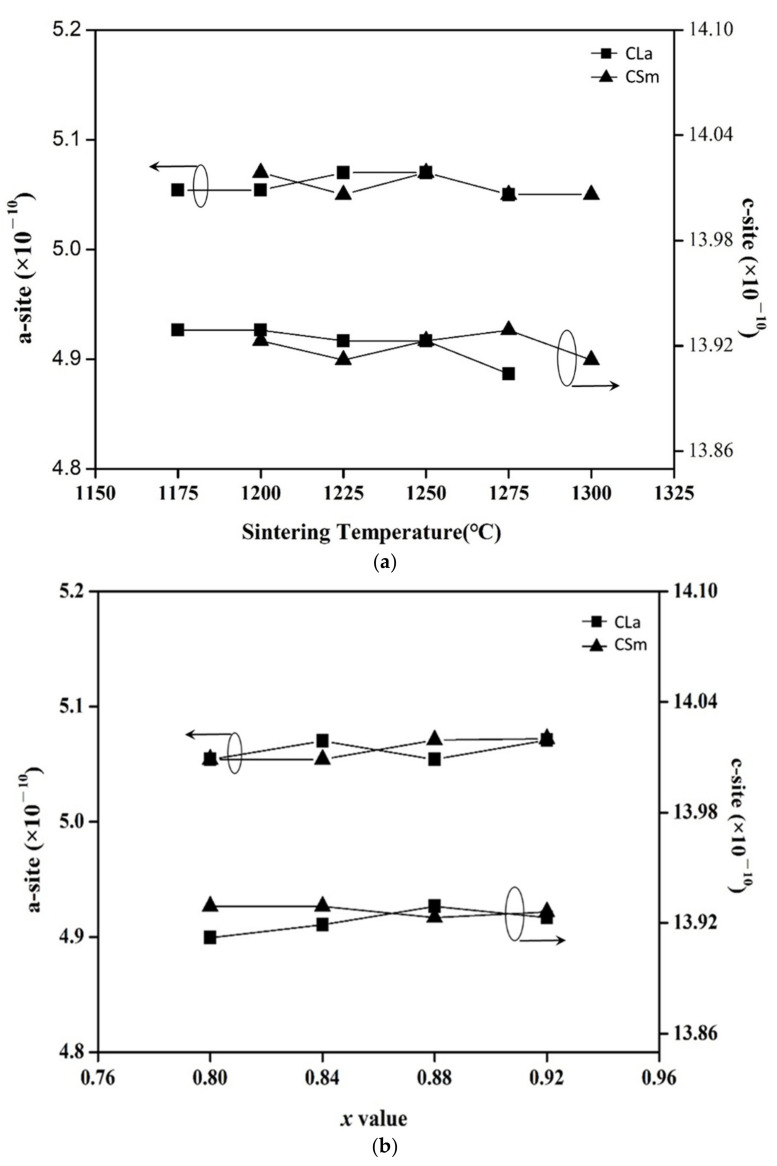
Lattice parameters of (**a**) 0.88(Mg_0.6_Zn_0.4_)_0.95_Ni_0__.__05_TiO_3_-0.12Ca_0__.6_La_0__.8__/3_TiO_3_/Ca_0__.8_Sm_0__.4__/3_TiO_3_ at various sintering temperature, (**b**) x(Mg_0.6_Zn_0.4_)_0.95_Ni_0__.__05_TiO_3_-(1 − x) Ca_0__.6_La_0__.8__/3_TiO_3_ sintered at 1225 °C and Ca_0__.8_Sm_0__.4__/3_TiO_3_ sintered at 1250 °C.

The lattice parameters of (Mg_0.6_Zn_0.4_)_0.95_Ni_0.05_TiO_3_ mixed phase ceramics as a function of sintering temperature and x value were also calculated, as shown in Figure 2a,b. A minor increase in both a-site and c-site was found for (Mg_0.6_Zn_0.4_)_0.95_Ni_0.05_TiO_3_ ceramics with the confronting of MgTiO_3_ (ICDD–PDF #00-006-0494). The consequences clarify that (Mg_0.6_Zn_0.4_)_0.95_Ni_0.05_TiO_3_ ceramics would compose a solid solution to replace Mg^2+^ with Zn^2+^. The lattice parameters vary from a = 5.054 Å and c = 13.898 Å of MgTiO_3_ [21] to a = 5.07 Å, and c = 13.923 Å with the formation of (Mg_0.6_Zn_0.4_)_0.95_Ni_0.05_TiO_3_[19]. The reason is that the ionic radii of Zn^2+^(0.82 Å) are much bigger than those of Mg^2+^ (0.72 Å). With the Ca_0.8_Sm_0.4/3_TiO_3_ and Ca_0.6_La_0.8/3_TiO_3_ additions, the lattice parameters of xMZNT-(1 − x)CLa/CSm ceramics don’t vary significantly with the increasing amounts of Ca_0.6_La_0.8/3_TiO_3_ and Ca_0.8_Sm_0.4/3_TiO_3_. This explanation proved the existence of a two phase system of xMZNT-(1 − x)CLa/CSm ceramics and strongly agreed with XRD patterns results shown in Figure 1.

**Figure 3:**
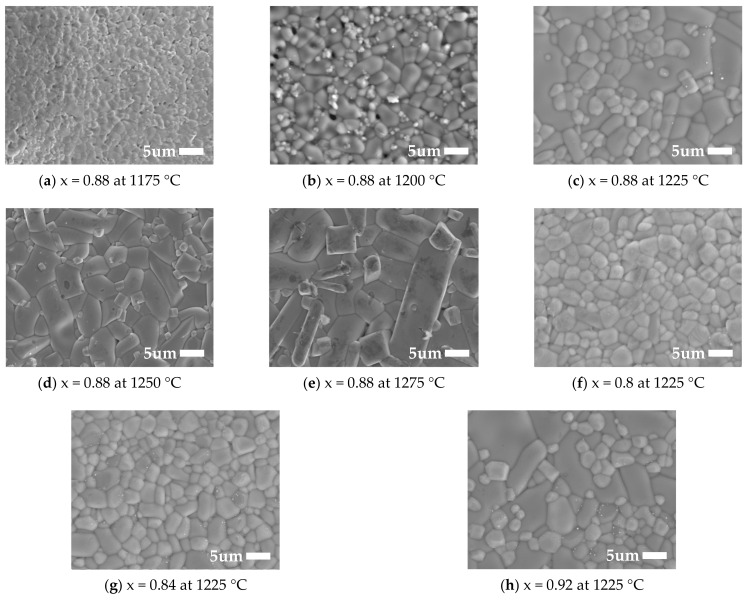
(**a**–**h**) Scanning electron microscopy photographs of Ca_0.6_La_0.8/3_TiO_3_-modified (Mg_0.6_Zn_0.4_)_0.95_Ni_0.05_TiO_3_ with x = 0.88 sintered from 1175 °C to 1275 °C for 4 h and with various x values sintered at 1225 °C for 4 h.

**Figure 4:**
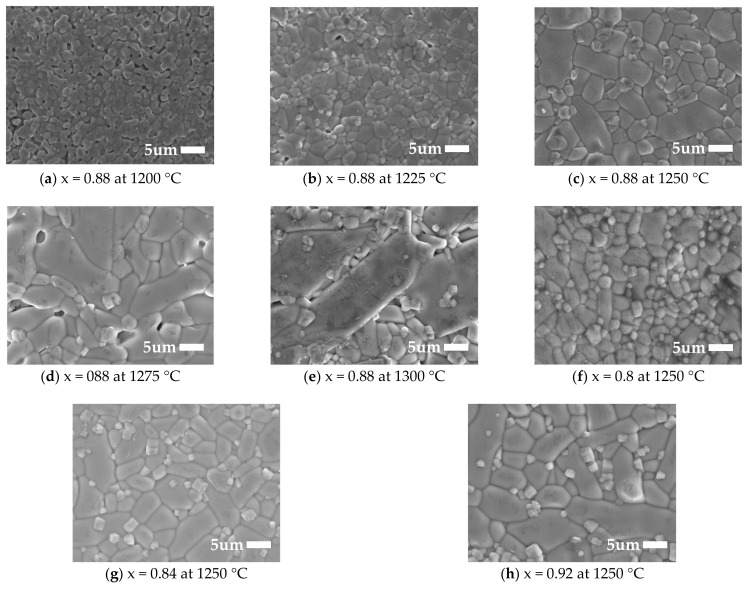
(**a**–**h**) Scanning electron microscopy photographs of Ca_0.8_Sm_0.4/3_TiO_3_-modified (Mg_0.6_Zn_0.4_)_0.95_Ni_0.05_TiO_3_ with x = 0.88 sintered from 1200 °C to 1300 °C for 4 h and with various x values sintered at 1250 °C for 4 h.

The microstructure photographs of xMZNT-(1 − x)CLa/CSm ceramics under x = 0.88 with different sintering temperatures and sintered at 1225 °C (Ca_0.6_La_0.8/3_TiO_3_)/1250 °C (Ca_0.8_Sm_0.4/3_TiO_3_) with varying values of x were revealed in Figure 3 and Figure 4, respectively. The average size of grains increased with the increasing sintering temperature, and microstructures revealed the most compact and the fewest pores at 1225 °C (Ca_0.6_La_0.8/3_TiO_3_)/1250 °C (Ca_0.8_Sm_0.4/3_TiO_3_). Moreover, the grain growth rate of (Mg_0.6_Zn_0.4_)_0.95_Ni_0.05_TiO_3_ was much more rapid than that of Ca_0.8_Sm_0.4/3_TiO_3_ or Ca_0.6_La_0.8/3_TiO_3_, which would result in a great size disparity in the specimens. This phenomenon exhibits that the existence of a Ca_0.8_Sm_0.4/3_TiO_3_ or Ca_0.6_La_0.8/3_TiO_3_ phase may repress irregular grain growth of the main phases, which supports the attainment of an excellent dielectric performance. However, excess amounts of Ca_0.6_La_0.8/3_TiO_3_/Ca_0.8_Sm_0.4/3_TiO_3_ contributed to the dielectric loss of the ceramics system, and high porosity may have directly affected the dielectric performances of the ceramic specimens. Furthermore, we also studied the specimens’ microstructures with x = 0.80–0.92 at the optimal sintering temperature for xMZNT-(1 − x)CLa/CSm under 1225 °C (Ca_0.6_La_0.8/3_TiO_3_)/1250 °C (Ca_0.8_Sm_0.4/3_TiO_3_). Generally speaking, well-densified samples with tiny porosity were obtained when samples sintered at 1225 °C (Ca_0.6_La_0.8/3_TiO_3_)/1250 °C (Ca_0.8_Sm_0.4/3_TiO_3_) with x = 0.80–0.92, but the surface morphology of the xMZNT-(1 − x)CLa/CSm varied significantly under x = 0.88 with a different sintering temperature.

**Figure 5:**
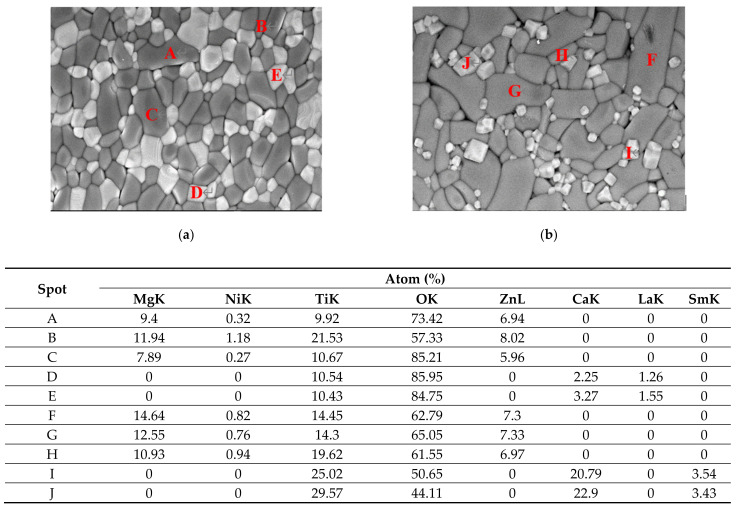
The BEI photograph and EDS results of (Mg_0.6_Zn_0.4_)_0.95_Ni_0.05_TiO_3_ ceramics with (**a**) Ca_0.6_La_0.8/3_TiO_3_ sintered at 1225 °C for 4 h, (**b**) Ca_0.8_Sm_0.4/3_TiO_3_ additions sintered at 1250 °C for 4 h.

Individual grain composition and distribution in the 0.88MZNT-0.12CLa/CSm ceramics sintered at 1225 °C (Ca_0.6_La_0.8/3_TiO_3_)/1250 °C (Ca_0.8_Sm_0.4/3_TiO_3_) were checked by EDS and the backscattered electronic image (BEI) as shown in Figure 5. The grains marked with spots A–J can be divided into three groups: huge dark grey polygons (spots A and C, and spots F and G), small bright grey polygons (spots D and E, and spots I and J), and small dark grey stick (spot B, and H). Huge polygons were distinguished as (Mg_0.6_Zn_0.4_)_0.95_Ni_0.05_TiO_3_ accompanying small polygons Ca_0.8_Sm_0.4/3_TiO_3_ or Ca_0.6_La_0.8/3_TiO_3_ crystallites nearby. The distributed small stick was indexed as (Mg_0.6_Zn_0.4_)_0.95_Ni_0.05_Ti_2_O_5_, which is not a dominant element in the specimen. As expected, xMZNT-(1 − x)CLa/CSm phases separated since they exhibited virtually no solubility between them due to different crystal structures. This discussion was further confirmed in BEI analysis.

**Figure 6:**
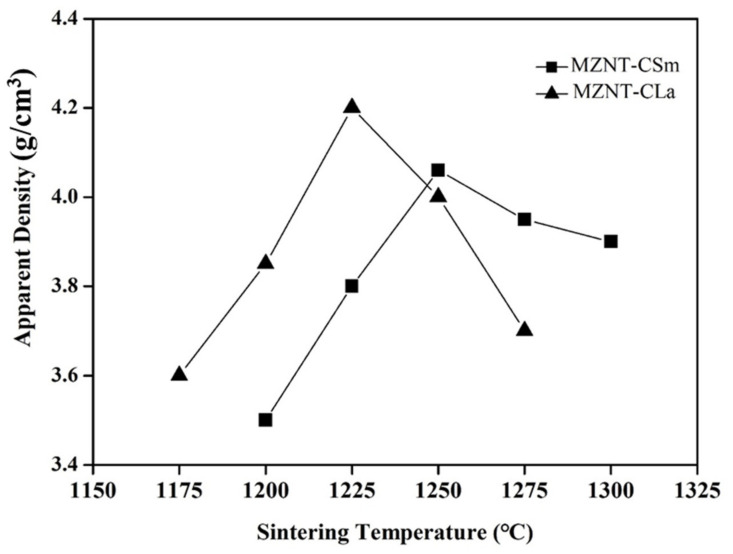
Dependence of apparent density on sintering temperature of the 0.88(Mg_0.6_Zn_0.4_)_0.95_Ni_0__.__05_TiO_3_-0.12Ca_0__.6_La_0__.8__/3_TiO_3_/Ca_0__.8_Sm_0__.4__/3_TiO_3_ ceramics.

Figure 6 shows the apparent densities of the xMZNT-(1 − x)CLa/CSm ceramics system sintered at various temperatures for 4 h. With the rise in sintering temperature, the apparent density reached a maximum value of 1225 °C (Ca_0.6_La_0.8/3_TiO_3_)/1250 °C (Ca_0.8_Sm_0.4/3_TiO_3_). This resulted from the ceramics’ microstructure being denser, as observed in Figure 3 and Figure 4. In addition, the apparent densities were also a function of the combinations and raised with the reducing x value due to the heavier nature of Ca_0.8_Sm_0.4/3_TiO_3_/Ca_0.6_La_0.8/3_TiO_3_ than (Mg_0.6_Zn_0.4_)_0.95_Ni_0.05_TiO_3_, as shown in Table 1.

### 2.2. Microwave Dielectric Properties

**Figure 7:**
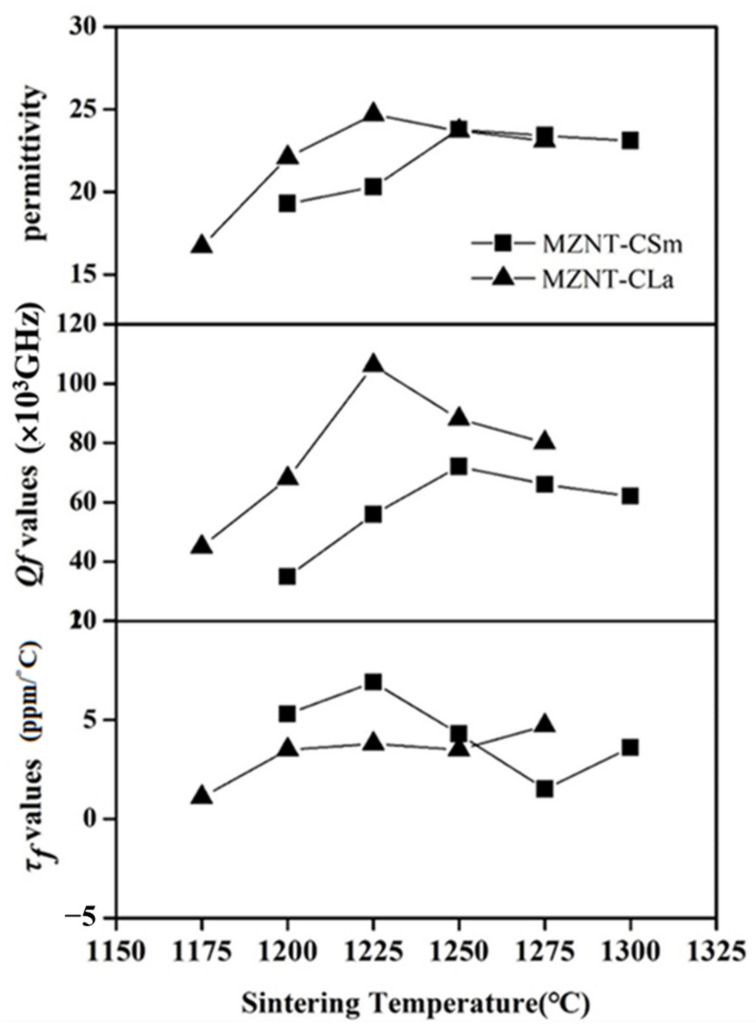
The dielectric properties of the 0.88(Mg_0.6_Zn_0.4_)_0.95_Ni_0__.__05_TiO_3_-0.12Ca_0__.6_La_0__.8__/3_TiO_3_/ Ca_0__.8_Sm_0__.4__/3_TiO_3_ ceramics as a function of the sintering temperature.

**Figure 8:**
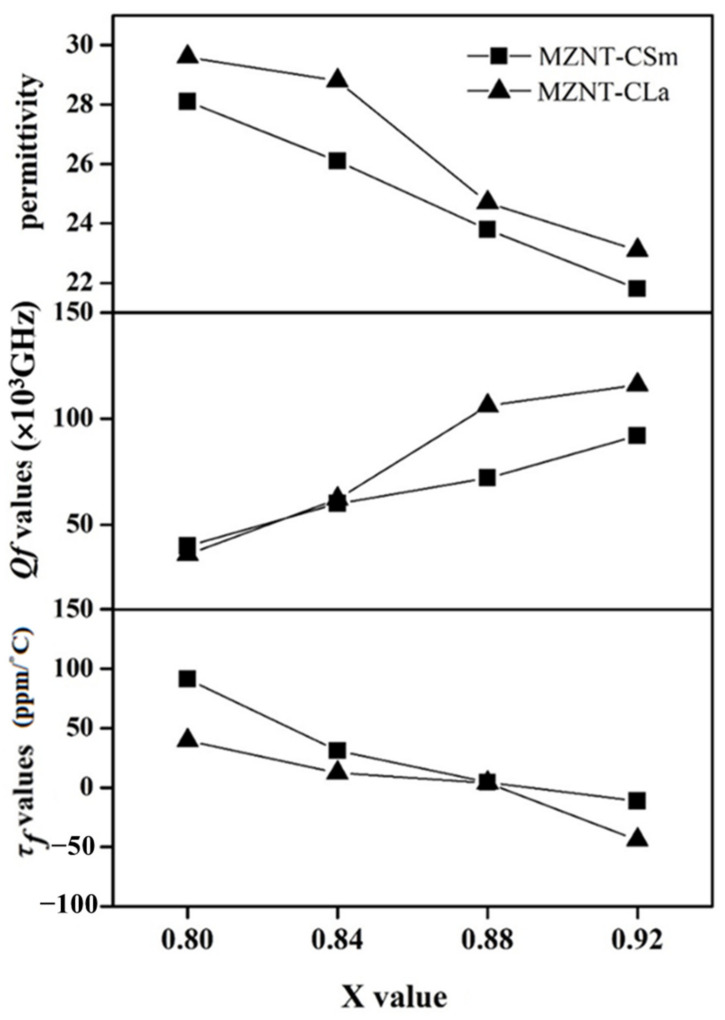
The dielectric properties of the x(Mg_0.6_Zn_0.4_)_0.95_Ni_0__.__05_TiO_3_-(1 − x)Ca_0__.6_La_0__.8__/3_TiO_3_ sintered at 1225 °C/Ca_0__.8_Sm_0__.4__/3_TiO_3_ sintered at 1250 °C as a function of the x values.

The tunable dielectric properties of the xMZNT-(1 − x)CLa/CSm ceramics as a function of the sintering temperature and x value were shown in Figure 7 and Figure 8, respectively. The correlation between permittivity (ε_r_) and sintering temperatures exhibited an equivalent tendency between densities and sintering temperatures, because higher density is physically equivalent to lower porosity. The permittivity slightly increased with the rising sintering temperature. The ε_r_ of the 0.88(Mg_0.6_Zn_0.4_)_0.95_Ni_0.05_TiO_3_-0.12Ca_0.6_La_0.8/3_TiO_3_ ceramics gradually vary from 19.3 to 23.8 as the sintering temperature ranged from 1175 °C to 1225 °C and, after that, decreased after 1250 °C. Furthermore, the dielectric performances of the xMZNT-(1 − x)CLa/CSm ceramics as a function of the x value were shown in Figure 8. The ε_r_ was raised with a reducing x value due to a higher permittivity (ε_r_) of Ca_0.6_La_0.8/3_TiO_3_ and Ca_0.8_Sm_0.4/3_TiO_3_ additions.

The quality factor is a significant symbol for the utilizations of dielectric ceramics at microwave frequency, since a higher quality factor means a lower dielectric loss for microwave frequency devices. The quality factor of (Mg_0.6_Zn_0.4_)_0.95_Ni_0.05_TiO_3_ is much higher than that of Ca_0.8_Sm_0.4/3_TiO_3_ and Ca_0.6_La_0.8/3_TiO_3._ Hence, it is supposed that the *Qf* values should reduce with the rising amount of Ca_0.6_La_0.8/3_TiO_3_/Ca_0.8_Sm_0.4/3_TiO_3_. The *Qf* values of xMZNT-(1 − x)CLa/CSm ceramics system reduce with the combination (x), as shown in Figure 8. The microwave dielectric loss is principally occasioned by the lattice vibrational modes, pores, and second phases [23]. The *Qf* value of xMZNT-(1 − x) CLa raised with the sintering temperature increased from 1175 °C to 1225 °C (maximum *Qf* at 1225 °C) and decreased gradually. The increment of *Qf* value at 1175 °C to 1225 °C was high relative to the density rise and the uniformity of grain growth, as observed in Figure 3 and Figure 4. At 1225 °C, the maximum *Qf* value of around 106,000 GHz was measured for the 0.88(Mg_0.6_Zn_0.4_)_0.95_Ni_0.05_TiO_3_-0.12 Ca_0.6_La_0.8/3_TiO_3_ ceramics. The downgrade in *Qf* value was attributed to inhomogeneous grain growth, and resulted in a reduction in density shown in Figure 3 and Figure 4. Since the *Qf* value of xMZNT-(1 − x)CLa/CSm ceramics was consistent with the variation of density, it implied that the dielectric loss of xMZNT-(1 − x)CLa/CSm ceramics was primarily dominated by the bulk density [24,25].

The resonant frequency temperature coefficient is strongly related to the mixture, the additions, and the second phase of a material [26]. For example, the τ*_f_* values of xMZNT-(1 − x)CLa/CSm ceramics rapidly improved with reducing x value due to τ*_f_* compensator Ca_0.6_La_0.8/3_TiO_3_/Ca_0.8_Sm_0.4/3_TiO_3_ additions. However, a significant change in the τ*_f_* value was not observed of specimens at different sintering temperatures; it only slightly varied from 1 to 7 ppm/°C as the temperature range remained below 100 °C. It also demonstrated a transition of τ*_f_* value from negative to positive as x varied from 0.92 to 0.80. Thus, a near-zero τ*_f_* value can be achieved by proper stoichiometric calculation.

Table 1 demonstrated the tunable microwave dielectric properties of xMZNT-(1 − x)CLa/CSm ceramic system. As the x value decreased from 0.92 to 0.80, the τ*_f_* values of xMZNT-(1 − x)CLa ceramics at 1225 °C ranged from −44.0 to +39.4 ppm/°C, and xMZNT-(1 − x)CSm ceramics at 1250 °C ranged from −11.3 to +91.2 ppm/°C. The more comprehensive τ*_f_* range for xMZNT-(1 − x)CSm mixture was due to the high value τ*_f_* compensator addition of Ca_0.8_Sm_0.4/3_TiO_3_ (~400 ppm/°C). We also consider *Qf* value at near-zero τ*_f_*, and xMZNT-(1 − x)CLa revealed a higher *Qf* value. Overall, a ceramic system with the following properties: low sintering temperature, high *Qf* value, mostly near-zero τ*_f_* values, etc., was regarded as a suitable dielectric mixture. Therefore, 0.88(Mg_0.6_Zn_0.4_)_0.95_Ni_0.05_TiO_3_-0.12 Ca_0.6_La_0.8/3_TiO_3_ sintered at 1225 °C/4 h with a permittivity (ε_r_) of 24.7, a *Qf* value of 106,000 GHz, and a *τ_f_* value of 3.8 ppm/°C was recommended as the potential candidate adopted in practical applications.

Table 2 describes the microwave dielectric properties of relative dielectrics and mixtures with τ*_f_* compensator. With Ca_0.6_La_0.2667_TiO_3_ addition, 0.88(Mg_0.6_Zn_0.4_)_0.95_Ni_0.05_TiO_3_-0.12 Ca_0.6_La_0.8/3_TiO_3_ demonstrated the 3.92% higher *Qf* and 8.2% lower thermal budget with comparable near-zero τ*_f_* than 0.85Mg_0.95_Ni_0.05_TiO_3_-0.15 Ca_0.6_La_0.8/3_TiO_3_. This improvement makes (Mg_0.6_Zn_0.4_)_0.95_Ni_0.05_TiO_3_-based ceramics a potential substrate material candidate for adoption in industrial applications. Surfing the applications of 0.88(Mg_0.6_Zn_0.4_)_0.95_Ni_0.05_TiO_3_-0.12 Ca_0.6_La_0.8/3_TiO_3_ in 5G wireless communications in the future is attractive.

## 3. Experimental Procedure

Traditional solid-state ceramic methods were utilized to synthesize samples of (Mg_0.6_Zn_0.4_)_0.95_Ni_0.05_TiO_3_, Ca_0.6_La_0.8/3_TiO_3_, and Ca_0.8_Sm_0.4/3_TiO_3_ from high-purity oxide powders (>99.9%): MgO, NiO, ZnO, CaCO_3_, La_2_O_3_, Sm_2_O_3_, and TiO_2_. First, the starting materials were mixed according to the stoichiometry: (Mg_0.6_Zn_0.4_)_0.95_Ni_0.05_TiO_3_, Ca_0.6_La_0.8/3_TiO_3_, and Ca_0.8_Sm_0.4/3_TiO_3_. Then, they were ground in distilled water for 24 h in a ball mill with agate balls. The mixed solution was dried in the oven and calcined at 1100 °C/4 h for (Mg_0.6_Zn_0.4_)_0.95_Ni_0.05_TiO_3_, 1100 °C/4 h for Ca_0.6_La_0.8/3_TiO_3_, and 1250 °C/3 h for Ca_0.8_Sm_0.4/3_TiO_3_ in a high-temperature furnace. The calcined reagents were mixed in the second step according to the formula of x(Mg_0.6_Zn_0.4_)_0.95_Ni_0.05_TiO_3_-xCa_0.6_La_0.8/3_TiO_3_/Ca_0.8_Sm_0.4/3_TiO_3_ and ground into a fine powder for 24 h. A 3.5 wt% of a 12% PVA solution as a binder (Polyvinyl alcohol 500, Showa) was added into the calcined powder, granulated by sieving through a 100 mesh, and pressed into pellets, 1.1 cm in diameter and 0.5 cm in thickness, under 200 MPa pressure. The pellets were sintered at temperatures ranging from 1175 °C to 1275 °C for 4 h in air. The heating and cooling rates of the high-temperature furnace were set at 10 °C/min to obtain high-quality samples.

The crystallization of the sintered bulks was checked by XRD using CuKα (*λ* = 0.15406 nm) with a Siemens D5000 diffractometer in the 2θ range from 20° to 80°. The lattice constant was calculated using software with the Rietveld method to fit the XRD patterns. [16] A.C. Larson, R.B. Von Dreele, Los Alamos Laboratory Report LAUR 86-748, Los Alamos National Laboratory, Los Alamos, NM, 1988. The microstructural observation of the sintered surface morphology was carried out using scanning electron microscopy equipped with energy-dispersive X-ray spectroscopy (EDS) and backscattered electronic image (BEI). The apparent densities of the sintered samples were measured using the Archimedes method. The *ε_r_* and *Qf* were measured using the Hakki–Coleman dielectric resonator methodology [27], as improved by Courtney [28]. This method utilizes parallel conducting plates and coaxial probes in TE_011_ mode. TE represented transverse electric waves. The first two subscript integers denote the waveguide mode, and the third integer subscript indicates the order of resonance in an increasing set of discrete resonant lengths. The measurement system was connected to an vector network analyzer with Anritsu’s model MS46122B (Atsugi, Japan). The τ*_f_* value was measured with an identical setup but in the thermostat ranging from 20 °C to 80 °C. The following formula was utilized to obtain τ*_f_* value (ppm/°C):

(2) $$\tau_{f}=\frac{f_{2}-f_{1}}{f_{1}\left(T_{2}-T_{1}\right)}$$

where *f*_1_ and *f*_2_ represent the resonance frequencies at *T*_1_ = 20 °C and *T*_2_ = 80 °C, respectively.

## 4. Conclusions

In this study, Ca_0.6_La_0.8/3_TiO_3_ and Ca_0.8_Sm_0.4/3_TiO_3_-modified (Mg_0.6_Zn_0.4_)_0.95_Ni_0.05_TiO_3_ ceramics were investigated to obtain a near-zero temperature coefficient with appropriate dielectric properties. It showed mixed phases of (Mg_0.6_Zn_0.4_)_0.95_Ni_0.05_TiO_3_ and Ca_0.8_Sm_0.4/3_TiO_3_ or Ca_0.6_La_0.8/3_TiO_3_ accompanied by second phase (Mg_0.6_Zn_0.4_)_0.95_Ni_0.05_Ti_2_O_5_. The permittivity and temperature coefficient values of x(Mg_0.6_Zn_0.4_)_0.95_Ni_0.05_TiO_3_-(1 − x) Ca_0.8_Sm_0.4/3_TiO_3_/Ca_0.6_La_0.8/3_TiO_3_ ceramics were controllable by adjusting the x value and the *Qf* value increase as the x value rose. It is worth noting that optimized 0.88(Mg_0.6_Zn_0.4_)_0.95_Ni_0.05_TiO_3_–0.12Ca_0.6_La_0.8/3_TiO_3_ ceramic systems possessed good microwave dielectric properties—a permittivity (ε_r_) of 24.7, a *Qf* value of 106,000 GHz, and a *τ_f_* value of 3.8 ppm/°C at 1225 °C/4 h—and so the system was considered an excellent candidate to fabricate substrates for wireless component applications in the future.

## Figures and Tables

**Table 1:** Microwave dielectric properties of (Mg_0.6_Zn_0.4_)_0.95_Ni_0.05_TiO_3_ with Ca_0.6_La_0.8/3_TiO_3_ sintered at 1225 °C, and Ca_0.8_Sm_0.4/3_TiO_3_ sintered at 1250 °C for 4 h.

**x(Mg** **_0.6_Zn_0.4_** **)** **_0.95_** **Ni**_**0.05**_ **TiO**_**3**_ **-(1 − x)Ca**_**0.6**_ **La**_**0.8/3**_ **TiO**_**3**_					
**x value**	**S.T.(°C)**	**Density (g/cm** **^3^)**	**ε** **_r_**	***Qf*** **(Hz)**	**τ** *_**f**_* **(ppm/°C)**
**0.92**	1225	4.0	23.1	116,000	−44.0
**0.88**		4.2	24.7	106,000	3.8
**0.84**		4.4	28.8	62,000	12.3
**0.80**		4.5	29.6	36,000	39.4
**x(Mg_0.6_Zn_0.4_)_0.95_Ni_0.05_TiO_3_-** **(1 − x)Ca_0.8_Sm_0.4/3_TiO_3_**					
**x value**	**S.T.(** **°** **C)**	**Density (g/cm^3^)**	**ε_r_**	***Qf* (Hz)**	**τ*_f_*(ppm/** **°C)**
**0.92**	1250	4.05	21.8	92,000	−11.3
**0.88**		4.06	23.8	72,000	4.3
**0.84**		4.10	26.1	60,000	30.9
**0.80**		4.40	28.1	40,000	91.2

**S.T.**: Sintering Temperature.

**Table 2:** Comparison of the proposed dielectric with other similar reported dielectric ceramics.

Composition	S.T.(°C)	Permittivity	*Qf* (Hz)	τ*_f_* (ppm/°C)	Ref
(Mg_0.95_Ni_0.05_)TiO_3_	1350 °C/4 h	17.35	192,000	−47.0	[13]
0.85(Mg_0.95_Ni_0.05_)TiO_3_-0.15Ca_0.6_La_0.8/3_TiO_3_	1325 °C/4 h	24.61	102,000	−3.6	[15]
(Mg_0.6_Zn_0.4_)_0.95_Ni_0.05_TiO_3_	1200 °C/4h	19.30	165,000	−65.4	[16]
0.88(Mg_0.6_Zn_0.4_)_0.95_Ni_0.05_TiO_3_-0.12Ca_0.6_La_0.8/3_TiO_3_	1225 °C/4 h	24.70	106,000	3.8	This work

## Data Availability

The data are contained within the article.

## Sample Availability

Samples of the compounds are not available from the authors.
